# The Impact of Previous Acute Decompensation on the Long-Term Prognosis of Alcoholic Hepatitis in Cirrhotic Patients

**DOI:** 10.3390/jcm8101600

**Published:** 2019-10-03

**Authors:** Eileen L. Yoon, Tae Yeob Kim, Do Seon Song, Hee Yeon Kim, Chang Wook Kim, Young Kul Jung, Dong Hyun Sinn, Jae Young Jang, Moon Young Kim, Soung Won Jeong, Sang Gyune Kim, Ki Tae Suk, Dong Joon Kim, on behalf of the Korean Acute-on-Chronic Liver Failure (KACLiF) Study Group

**Affiliations:** 1 Department of Internal Medicine, Inje University College of Medicine, Seoul 01757, Korea; mseileen80@gmail.com; 2 Department of Internal Medicine, New Hope Internal Medicine Clinic, Seoul 03113, Korea; ktydoc@hanmail.net; 3 Department of Internal medicine, The Catholic University of Korea, Seoul 06591, Korea; dsman@catholic.ac.kr (D.S.S.); hee82@catholic.ac.kr (H.Y.K.); cwkim@catholic.ac.kr (C.W.K.); 4 Department of Internal Medicine, Korea University, Seoul 02841, Korea; 93cool@hanmail.net; 5 Department of Internal Medicine, Samsung Medical Center, Seoul 06531, Korea; sinndhn@hanmail.net; 6 Department of Internal Medicine, Soonchunhyang University, Seoul 04401, Korea; jyjang@schmc.ac.kr (J.Y.J.); jeongsw@schmc.ac.kr (S.W.J.); 7 Department of Internal Medicine, Yonsei University Wonju College of Medicine, Wonju 26426, Korea; drkimmy@yonsei.ac.kr; 8 Department of Internal Medicine, Soonchunhyang University, Bucheon 14584, Korea; mcnulty@schmc.ac.kr; 9 Department of Internal Medicine, Hallym University College of Medicine, Institute for Liver and Digestive Diseases, Hallym University, Chuncheon 24253, Korea; diarrhea100@hanmail.net

**Keywords:** alcoholic hepatitis, liver cirrhosis, acute decompensation, survival, mortality

## Abstract

Recurrent episodes of liver injury may either waste hepatic reserve or induce tolerance to further injury. We aimed to investigate whether the previous acute decompensation (AD) in liver cirrhosis (LC) affects the long-term transplant-free survival of patients with alcoholic hepatitis (AH). The survival data of 894 alcoholic LC cohort who had been admitted with acute deterioration in 21 academic hospitals in Korea were prospectively followed up. Enrolled patients were divided into three groups: Group 1, without AH; group 2, with nonsevere AH; and group 3, with severe AH. Although the baseline liver function was not different between the groups with or without previous AD, it was a significant predictor of poor long-term outcomes. The presence of previous AD negatively affected long-term overall survival (HR 1.62, 95% C.I. 1.20–2.18, *p* = 0.002) in groups 1 and 2 as a whole, independent of the Model for End-stage Liver Disease score. The three-month conditional survival was significantly worse in group 3 for up to 12 months in the presence of previous AD (*p* < 0.05). We concluded that not only the severity of AH, but also the prior AD is an important predictor of long-term outcomes in alcoholic LC patients with acute deterioration.

## 1. Introduction

Alcoholic hepatitis (AH) is a clinical syndrome distinguished by a recent onset of jaundice with an increased risk of liver-related events [1]. AH can occur in any stage of alcoholic liver disease; however, most patients with severe AH already have coexisting liver cirrhosis (LC) [2]. A potential commonality between severe AH and LC is that previous experience of acute decompensation (such as over ascites, hepatic encephalopathy, and gastrointestinal bleeding) may be an important prognostic factor for the natural course of AH, as it is in alcohol-related (alcoholic) LC. Also, drawing a line between the patient who had or had not suffer the previous acute decompensation (AD) can be an important issue in the progression of LC that a patient can be in the process of decompensation or can be already in a decompensated state.

Meanwhile, systemic inflammatory response syndrome (SIRS) often complicates severe AH, leading to multi-organ failure and a poor short-term outcome [2,3,4]. Short-term mortality reaches 35–50% without proper treatment or with a null response to corticosteroids [5]. The baseline Model for End-Stage Liver Disease (MELD) score and Lille model were significant factors for the short-term prognosis of severe AH patients [6]. However, the MELD score and Lille model do not consider the development or severity of organ failure other than liver and kidney failures, and these other organ failures can also significantly affect the short-term mortality of severe AH patients. 

According to our previous study on long-term outcome of acute-on-chronic liver failure (ACLF), we can assume that it is similar to that of chronic liver disease in that they are both affected by a history of AD prior to the index events [7]. A potential commonality between severe AH and ACLF is that previous AD history may also be an important prognostic factor affecting long-term outcomes of severe AH, as it is in ACLF. 

Additionally, alcoholic LC patients suffer recurrent episodes of AH regardless of severity. Recurrent episodes of liver injury may either waste the hepatic reserve or induce tolerance to further liver injury. Furthermore, data regarding long-term prognosis or prognostic factors of AH other than alcohol abstinence are limited compared with data regarding the short-term prognosis of AH [6,8]. 

Therefore, we aimed to investigate the long-term prognosis and factors (including a previous history of AD) associated with the long-term outcome of alcoholic LC patients.

## 2. Experimental Section

### 2.1. Patients

**Figure 1:**
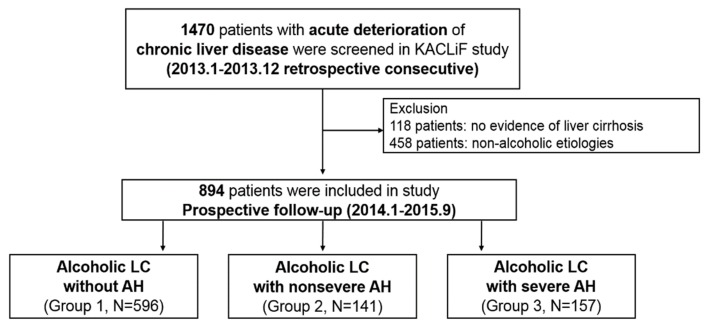
Flowchart of patients enrolled in the study. AH, alcoholic hepatitis; KACLiF, Korean Acute-on-Chronic Liver Failure; LC, liver cirrhosis; N, number.

Alcoholic LC patients from the retrospective Korean Acute-on-Chronic Liver Failure (KACLiF) cohort were included in this study [9]. The KACLiF cohort, despite its nickname, consisted of 1470 patients who were admitted due to acute deterioration of either chronic liver disease or LC between January 2013 and December 2013 at 21 nationwide academic hospitals in Korea. The cohort also included patients who developed ACLF at admission or afterward (Figure 1). Patients with hepatocellular carcinoma or liver disease from coexisting causes other than excessive alcohol consumption were excluded. In the KACLiF study, AD was defined as having one or more conditions, such as overt ascites, hepatic encephalopathy, gastrointestinal bleeding (including variceal bleeding), and bacterial infection. Furthermore, “acute deterioration”, with a broader concept of AD, was defined as having one or more features of AD and/or hyperbilirubinemia (serum bilirubin ≥ 3 mg/dL). We included patients with hyperbilirubinemia per the inclusion criteria to ensure that all patients who might be adequate for inclusion were included. Admission due to reasons stated above could be objective events which can be caught in a retrospective review of electronic medical charts and laboratory data.

Of the initial 1470 patients from the KACLiF cohort, 118 patients without evidence of LC and 458 patients with non-alcoholic etiologies were excluded. LC was defined based on the baseline features of the radiologic findings on ultrasonography or computed tomography scans (e.g., undulating liver surface, compensatory left lobe hypertrophy, splenomegaly, and portosystemic shunts) and/or based on various clinical findings, including: (1) thrombocytopenia (<150,000/mm^3^), (2) the presence of splenomegaly, (3) the presence of ascites, and (4) the presence of esophageal or gastric varix from endoscopy. 

Patients were subdivided into three groups according to the presence and severity of AH (group 1: Alcoholic LC with acute deterioration other than AH; group 2: Alcoholic LC with acute deterioration and nonsevere AH; and group 3: Alcoholic LC with acute deterioration and severe AH). Patients with excessive alcohol consumption within the last two months (>60 g/day for males and >40 g/day for females), serum bilirubin ≥ 3 mg/dL, and aspartate aminotransferase to alanine aminotransferase ratio > 1.5 were considered to have AH [10]. Severe AH was defined as having a modified discriminant function (DF) score of ≥ 32. Patients with severe AH were treated with either prednisolone or pentoxifylline, and the decision was left to the physician’s discretion. Survival data of these patients were prospectively collected until September 2015 (Supplementary Figure S1). 

Patients who were lost during follow-up or underwent liver transplantation (LT) were censored. The missing survival data were minimized by reconfirming the death of a patient via online contact with the patients or their families. This study was approved by the Institutional Review Board of all the centers participating in this study.

### 2.2. Study Endpoints

The primary endpoint of this study was to assess the long-term transplant-free survival of alcoholic LC patients with acute deteriorating events. The secondary endpoint was to identify the factors that affect long-term transplant-free survival in alcoholic LC patients with acute deterioration.

### 2.3. Statistical Analysis

Categorical variables were expressed as number of patients with percentages (*n* (%)) and were analyzed using a *χ*^2^ test or Fisher’s exact test. Continuous variables were expressed as the mean ± standard deviation and were analyzed by Student’s independent *t*-test. The transplant-free overall survival was calculated with the Kaplan-Meier method using a log-rank test. To assess the prognostic predictors affecting long-term transplant-free survival after acute deteriorating events in alcoholic LC patients, the variables, including sex, age, presence of previous AD history, Child-Turcotte-Pugh (CTP) score, MELD score, and chronic liver failure-sequential organ failure assessment (CLIF-SOFA) score, were used in the Cox regression models and hazard ratios (HRs) of independent predictive factors. Variables that were found to be significant via univariate analysis were further analyzed using Cox multivariate regression. To exclude the effects of multicollinearity in multivariate analysis, we evaluated CTP in Model one and MELD in Model two, respectively. Statistical significance was set at a *p*-value less than 0.05. Statistical analysis was performed using SPSS 21.0 software (SPSS, Inc. an IBM Company, Chicago, IL, USA).

### 2.4. Conditional Survival Estimates 

Conditional survival is defined as the probability of surviving an additional number of months (y) given that a patient has survived for x number of months after diagnosis [11]. When S(*t*) represents the Kaplan-Meier survival estimates at time t, conditional survival can be calculated as S(y|x) = S(x + y)/S(x) [12]. Apart from the overall survival, which estimates prognosis at the time of diagnosis, the probability of survival dynamically evolves over time when patients receive intensive treatment for acute events. For example, the probability of surviving an additional three months (i.e., six months) in patients who had survived for three months after acute deterioration is calculated as S(6):S(3|3) = S(3 + 3)/S(3). Conditional survival estimates can be calculated from Kaplan-Meier survival estimates and the actuarial life table. The conditional Cox regression model was fitted by applying the traditional Cox regression method to the data set consisting of patients who have survived for different lengths of time (i.e., 3-, 6-, 9-, 12-, 15-, and 18-months) [12]. Conditional survival was compared with overall survival using the variable determined by multivariate analysis to be independent risk factors for survival.

## 3. Results

### 3.1. Baseline Characteristics

Of the 894 alcoholic LC patients with acute deteriorating events included in this study, 747 patients were male (83.6%) (Table 1). The mean age of these patients was 53 years old. No patients had other coexisting causes of liver injury, such as viral hepatitis. Nearly half of the patients had a previous history of AD. Their mean MELD and modified DF mean scores were 17 and 28, respectively. The numbers of patients in groups 1, 2, and 3 were 596, 141, and 157, respectively. The mean ages of patients with AH, regardless of severity, were lower than those of patients with only acute deteriorations other than AH (group 1). The proportions of patients with a previous history of AD were lower in groups 2 and 3 than in group 1. Patients in group 3 with severe AH showed more significant features of systemic inflammatory reaction or organ failure, such as elevated levels of white blood cells, creatinine, and higher CLIF-SOFA scores compared to those of groups 1 and 2. Of group 3, 13% and 10% were treated with corticosteroid and pentoxifyllin for severe AH, respectively. According to the types of acute deterioration at admission, the rates of ascites and bacterial infection were higher, and the rates of gastrointestinal bleeding were lower in group 3 than in groups 1 and 2.

### 3.2. Long-Term Prognosis of Acutely Deteriorated Alcoholic LC Patients

In the entire cohort, 263 patients (29.4%) died or underwent LT during the follow-up: 155 out of 596 patients (26.0%) in group 1, 27 out of 141 patients (19.1%) in group 2, and 75 out of 157 patients (47.8%) in group 3. The mean duration of the follow-up period was 14.3 ± 10.7 months. The three most common causes of death were gastrointestinal bleeding including variceal bleeding (*n* = 100, 38.0%), bacterial infection (*n* = 51, 19.4%), and hepatorenal syndrome (*n* = 31, 11.8%). A total of six patients underwent LT. They were five patients in group 1 and one patient in group 3. Their median transplant-free survival was 7.6 months (range, 0.8–26.9). Eighty patients were lost to follow-up during the period. 

**Figure 2:**
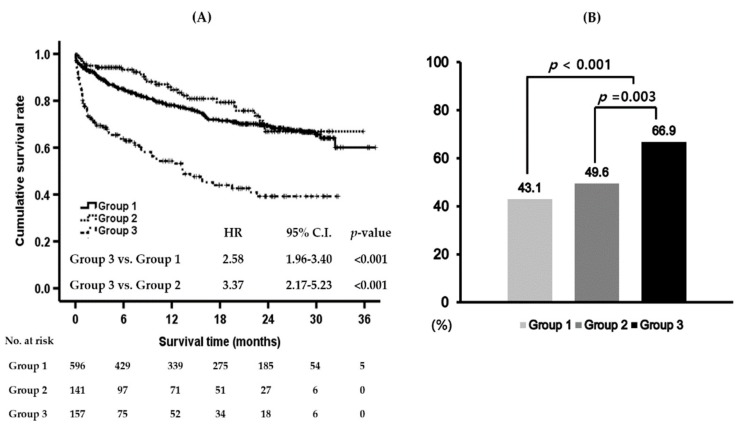
Long-term prognosis of alcoholic liver cirrhosis (LC) patients who were admitted with acute deterioration. (**A**) Patients who had experienced severe alcoholic hepatitis (AH) (group 3) showed the poorest long-term transplant-free overall survival among these groups. In the Cox regression analysis, the hazard ratios (HRs) of severe AH for long-term mortality were 2.58 (95% C.I. 1.96–3.40, *p* < 0.001) and 3.37 (95% C.I. 2.17–5.23, *p* < 0.001) compared to those of groups 1 and 2, respectively. The transplant-free overall survival was calculated with the Kaplan-Meier method using a log-rank test. (**B**) One-year mortalities of groups 1, 2, and 3 were compared in the entire cohort. Group 3 showed significantly higher one-year mortality than group 1 (*p* < 0.001) and group 2 (*p* = 0.003). AH, alcoholic hepatitis; C.I., confidence interval; HR, hazard ratio; LC, liver cirrhosis; No, number.

The mean overall survival in these groups were as followed: 27.6 months (95% confidence interval (C.I.) 26.3–28.9) in group 1, 28.4 months (95% C.I. 26.0–30.8) in group 2, and 16.6 months (95% C.I. 14.2–19.1) in group 3. Comparing the transplant-free overall survival among these groups, group 3 had significantly poorer survival than group 1 and group 2. However, survival was not different between group 1 and group 2 (Figure 2A). The HRs for long-term transplant free-survival of severe AH patients (group 3) were 2.58 (95% C.I. 1.96–3.40, *p* < 0.001) compared to that of group 1 and 3.37 (95% C.I. 2.17–5.23, *p* < 0.001) compared to that of group 2 (Figure 2A). Group 3 showed the highest one-year mortality (Figure 2B). 

### 3.3. Significant Predictors of Long-Term Transplant-Free Mortality in Alcoholic LC Patients with Acutely Deteriorating Events

Sex, age, and previous history of ADs as well as CTP, MELD, and CLIF-SOFA scores were evaluated to be possible predictors of long-term transplant-free mortality in alcoholic LC patients who experienced acute deteriorating events (Table 2). In the univariate analysis, the presence of previous AD history, CTP, MELD, and CLIF-SOFA scores were determined to be significant factors for the prediction of mortality. For the multivariate analysis, Model one and Model two consisted of previous AD history and either a combination of CTP and CLIF-SOFA or MELD and CLIF-SOFA scores. In these two multivariate analysis models, a history of previous AD was a significant predictive factor for long-term transplant-free mortality, independent of either the combination of baseline liver function (CTP and MELD scores) or organ failure (CLIF-SOFA score).

### 3.4. Comparison of Long-Term Transplant-Free Overall Survival among the Groups According to the Presence of Previous AD History

**Figure 3:**
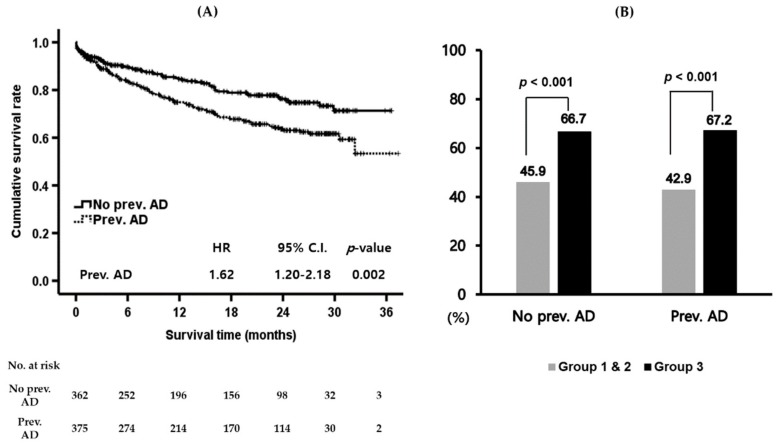
Long-term transplant-free overall survival of patients according to the presence of a previous AD history. (**A**) Long-term transplant-free overall survival was significantly different between the two subgroups according to the presence of a previous AD history in groups 1 and 2 as a whole (HR 1.62, 95% C.I. 1.20–2.18, *p* = 0.002). (**B**) Regarding one-year mortality, group 3 showed a poorer outcome compared to group 1 & 2 regardless of a previous AD history (*p* < 0.001). AD, acute decompensation; C.I., confidence interval; HR, hazard ratio; No., number.

As patients in group 2 showed similar results regarding long-term transplant-free overall survival compared with patients in group 1, groups 1 and 2 were analyzed as one group (groups 1 & 2). The long-term transplant-free overall survival of patients in groups 1 & 2 was significantly different according to the presence of a previous AD history (Figure 3A). The mean overall survival was 26.2 (24.5–27.9) months and 29.6 (95% C.I. 28.1–31.0) months in patients with and without previous AD (*p* = 0.001). The HR for previous AD history was 1.62 (95% C.I. 1.20–2.18, *p* = 0.002) in groups 1 & 2. Patients with previous AD history showed a higher prevalence of hepatic encephalopathy and gastrointestinal bleeding. However, sex, age, CTP, MELD, and CLIF-SOFA at admission were not different according to the presence or absence of previous AD history (Table 3).

Meanwhile, the long-term transplant-free overall survival of patients in group 3 was not different regardless of the previous AD history (Figure not shown). The one-year mortalities were higher in group 3 than in groups 1 & 2 regardless of the previous history of AD (both *p*-values less than 0.001) (Figure 3B).

### 3.5. Reversibility of Severe AH in Terms of Long-Term Transplant-Free Survival

In the entire cohort, regardless of previous AD history, the mean transplant-free overall survival of groups 1 & 2 and group 3 was 27.9 months and 16.6 months, respectively, and they were significantly different (HR of group 3: 2.73, 95% C.I. 2.09–3.57, *p* < 0.001). To offset the effects of short-term mortalities, we compared the mean transplant-free survival of patients who survived more than three months, six months, and 12 months after acute deteriorating events as a landmark estimation [13]. The impact of severe AH in patients who were overcoming acute deteriorating events lasted for more than three months up to 12 months in the entire cohort (Table 4). 

**Figure 4:**
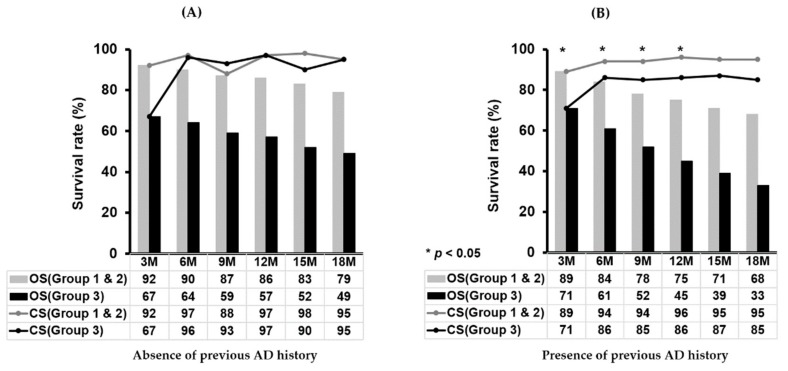
Rates of overall survival and conditional survival in groups 1 & 2 and group 3 regarding the presence of previous AD history. (**A**) In the absence of previous AD history, overall survival rates (bar graphs in black and gray) decreased over time in both groups and were significantly poorer in group 3 compared to groups 1 & 2 (*p* < 0.001). However, the three-month conditional survival rates (line graphs in black and gray) were not different between the two groups after acute deteriorating events. (**B**) In the presence of previous AD history, the overall survival rate in group 3 was significantly poorer than that in groups 1 & 2 (bar graphs in black and gray, *p* < 0.001). The three-month conditional survival rates of group 3 (line graph in black) were significantly lower than those of groups 1 & 2 (line graph in gray) up to 12 months after acute deteriorating events (*p* < 0.05). Asterisks depict that *p* values were less than 0.05 at each time point. AD, acute decompensation; C.I., confidence interval; CS, conditional survival; HR, hazard ratio; M, months; OS, overall survival.

In patients without a previous history of AD, the mean transplant-free overall survival of groups 1 & 2 and group 3 were 29.6 months and 18.1 months, respectively, and they were significantly different (HR of group 3: 3.18, 95% C.I. 2.16–2.49, *p* < 0.001). However, in subgroups of patients who survived more than three months, six months, and 12 months after acute deteriorating events, the mean transplant-free survival of group 3 was not significantly different from that of groups 1 & 2 (Table 4). Likewise, the three-month conditional survival rates were not different between the two groups (represented as black and gray line graphs) over the time after the onset of acute deteriorating events (Figure 4A).

In patients with a previous history of AD, the mean transplant-free overall survival of group 3 was much shorter than that of groups 1 & 2, which was 26.2 months and 14.2 months in groups 1 & 2 and group 3, respectively (HR for group 3: 2.64, 95% C.I. 1.80–3.88, *p* < 0.001). In the subgroups of patients who had a previous AD history and survived more than three months, six months, and 12 months after acute deteriorating events, the mean transplant-free overall survival of group 3 was significantly poorer than that of groups 1 & 2 (Table 4). Additionally, the three-month conditional survival estimates of group 3 (black line graph) remained less than that of groups 1 & 2(gray line graph) up to 12 months after acute deteriorating events (Figure 4B).

## 4. Discussion

To date, there has been much interest in the treatment options and prognostic factors for the short-term mortality of severe AH patients. However, data regarding the natural history and long-term prognosis of alcoholic LC patients are limited. Patients with alcoholic LC suffer recurrent episodes of acute deteriorating events, including jaundice, overt ascites, hepatic encephalopathy, gastrointestinal bleeding, and bacterial infection due to repeated alcoholism. According to one of the concepts from the The European Association for the Study of the Liver-chronic liver failure Acute-on-Chronic Liver Failure in Cirrhosis (CANONIC) study, of which nearly half of the cohort had alcoholic cirrhosis, decreased tolerance to inflammatory responses appears to be more marked in patients without previous AD than in patients with previous AD [14]. However, in our previous study, decreased hepatic reserve due to a previous history of AD appears to have a negative impact on the long-term outcomes of patients who survive ACLF [7]. Whether episodes of acute deteriorating events or severe AH waste hepatic reserve or induce tolerance in patients with alcoholic LC is not known. Additionally, whether experience with severe AH still affects long-term outcomes in patients who survive episodes of severe AH is unknown. Therefore, we analyzed the long-term overall survival and conditional survival of acutely deteriorated alcoholic LC patients.

The one-year mortality of severe AH reached 66.9% in our cohort. The long-term survival of patients was similar between group 1 and group 2, although the patients in group 1 may have had more advanced LC according to the higher prevalence of gastrointestinal bleeding and hepatic encephalopathy in comparison of AD at baseline. We found that a previous history of AD was a significant factor for mortality in alcoholic LC patients with acute deteriorating events independent of baseline hepatic function. The impact of a previous AD history was significant in groups 1 & 2. Additionally, it should be noted that those patients with or without a previous AD history do not display a significant gap in the CTP and MELD scores in groups 1 & 2 at baseline. Meanwhile, the effect of a previous AD history on long-term transplant-free overall survival was negligible in the cases of severe AH (group 3). Patients with severe AH had a lower prevalence of previous AD history than patients with acute deterioration other than AH according to the baseline characteristics. This could represent the decreased tolerance of the liver to the host inflammatory response in the setting of severe AH without a previous history of AD [14].

Interestingly, the three-month conditional survival of patients in group 3 could catch up to the conditional survival of patients in groups 1 & 2 in the absence of a previous AD history. However, the impact of severe AH was still significant considering conditional survival estimates in those with a previous AD history. There is no consensus regarding the definition of “severe AH recovery”. Traditionally, six months has been considered a minimum requirement of LT in alcoholic liver disease patients. Six months was adopted to provide time for the acute inflammatory reactions of recent alcohol intoxication to recover rather than to serve as proof of future abstinence [15]. Previously, we arbitrarily defined three months as the time for recovery of ACLF [7]. However, three months has been deemed to be too short a timeframe for recovery from severe AH events based on our finding that experience with severe AH could affect long-term transplant-free survival for up to 12 months in patients with a previous history of AD. In our study, we could assume that the characteristics of severe AH match those of acute-on-chronic liver failure in patients without a previous AD history by reversibility [16,17]. However, severe AH seems to act as a long-lasting deteriorating event that wastes hepatic reserve in patients with a previous AD history up to 12 months. This could be one facet of the evidence supporting early consideration of LT in medically nonresponding severe AH patients, especially when they have a previous history of AD. Commonly, severe AH with a prior history of decompensating events is considered an exclusion criterion for LT because of the high rate of alcohol relapse [18]. Our conflicting result that the impact of severe AH in long-term transplant-free survivors was not the same according to the presence of previous AD suggests that a cautious approach is needed when deciding to perform LT in highly selected severe AH patients with a previous history of AD. In our study, there were few people who were offered LT especially in group 3. The explanations for this low number of LT would be the inability to offer LT due to active alcoholism and characteristics of participating centers, which have lower access to LT regarding socioeconomic status and facilities.

There are several limitations to our study. First, although we collected prospective survival data, our data were originally based on a retrospective cohort. Therefore, we could not prove recovery of severe AH with serial laboratory findings, such as improvement in MELD scores. However, we analyzed the conditional survival estimate to confirm the impact of severe AH experience on the long-term outcome according to previous AD history. Second, as the mean follow-up period was 14.3 months, the difference in conditional survival between groups 1 & 2 and group 3 in the time beyond 12 months was not significant. To prove whether the hepatic reserve recovers after severe AH in the presence of previous AD requires a longer follow-up period for survival data. Third, we used the survival data of alcoholic LC patients (groups 1 & 2) as a reference group, but they were not at zero risk for being admitted for acute deteriorating events with or without nonsevere AH. Nevertheless, we can assume that the risk of previous AD history and severe AH is much higher than that of the reference group with more than minimal risk. Fourth, AH, especially severe AH was clinically diagnosed and in most cases in our study, diagnosis was not proven histologically. There would be a chance of over-diagnosis in group 3 which reflects the real-life practice in patients with active alcoholism. However, more than 90% of patients can be properly diagnosed with AH without liver biopsy [10]. Further, less than 10% of patients might have liver diseases other than AH, and thus this may have little influence on the results of our study. Fifth, the treatment of severe AH, the status of continued alcohol consumption, and socioeconomic status were not prospectively followed up, although the treatment response to steroid treatment, alcohol relapse, better support from their families are known to be the main independent predictive factors of short-term and long-term mortality [6,7]. It is possible that group 3 might have included the patients with the most alcohol use before enrolment due to their poor prognosis. However, the patients in group 3 were also the youngest among the three groups and had the lowest prevalence of previous AD history in our cohort, suggesting that the probability of selection bias for alcohol relapse in group 3 would be low. Additionally, the rate of alcohol relapse after LT is reported to be up to 50% in the first five years of follow up [19,20]. We can assume that the alcohol relapse rate might be similar among the three groups regardless of the presence and severity of AH, although it cannot be confirmed in our observational study. Apart from the long-term follow up of recidivism, the poorer prognosis of severe AH patients is well-known based on other studies and the alcohol relapse rates in these groups would not act as a confounding factor.

## 5. Conclusions

In conclusion, the long-term transplant-free survival of severe AH patients shows slower recovery in the presence of a previous AD history. Therefore, not only the severity of AH, but also the prior experience of AD is an important factor for the prediction of long-term outcomes in alcoholic LC patients with acute deterioration. Regarding the long-term prognosis of severe AH, better scoring systems considering a previous history of AD should be developed to include diverse subgroups of alcoholic LC patients. Preventing the first AD with interventions for alcohol abstinence is also important for the management of alcoholic LC patients. 

## Abbreviations

AHalcoholic hepatitisSIRSsystemic inflammatory response syndromeDFdiscriminant functionADacute decompensationCTPChild-Turcotte-PughC.I.Confidence intervalLCliver cirrhosisACLFacute-on-chronic liver failureMELDmodel for end-stage liver diseaseKACLiFKorean Acute-on-chronic liver failureCLIF-SOFAchronic liver failure-sequential organ failure assessmentHRshazard ratios

## Figures and Tables

**Table 1:** Baseline characteristics of the patients at admission.

	Total (*n* = 894)	Group 1 (*n* = 596)	Group 2 (*n* = 141)	Group 3 (*n* = 157)
Male^†^	747 (83.6%)	514 (86.2%)	116 (82.3%)	117 (74.5%)^§^
Age (years)	53 ± 10	55 ± 9	50 ± 9^‡^	48 ± 8^§^^¶^
Previous AD^†^	436 (48.8%)	317 (53.2%)	58 (41.1%)^‡^	61 (38.9%)^§^
White blood cells(×1000/mm^3^)	8.8 ± 5.2	8.4 ± 4.8	8.5 ± 5.1	10.3 ± 6.7^§^^¶^
Platelets (×1000/mm^3^)	109 ± 63	108 ± 57	114 ± 70	106 ± 73
Creatinine (mg/dL)	1.2 ± 1.3	1.2 ± 1.1	1.0 ± 1.2	1.5 ± 1.9^§^^¶^
Sodium (mEq/L)	135 ± 6	136 ± 6	134 ± 6^‡^	133 ± 7^§^
Types of AD				
Ascites^†^	301 (33.7%)	159 (26.7%)	66 (46.8%)^‡^	76 (48.4%)^§^
Hepatic encephalopathy^†^	141 (15.8%)	103 (17.3%)	13 (9.2%)^‡^	25 (15.9%)
Gastrointestinal bleeding^†^	390 (43.6%)	332 (55.7%)	29 (20.6%)^‡^	29 (18.5%)^§^
Infection^†^	82 (9.2%)	48 (8.1%)	13 (9.2%)	21 (13.4%)^§^
Child-Turcotte-Pugh score	9 ± 2	9 ± 2	9 ± 1^‡^	11 ± 1^§^^¶^
MELD score	17 ± 7	15 ± 6	17 ± 4^‡^	26 ± 7^§^^¶^
CLIF-SOFA score	5 ± 3	5 ± 3	5 ± 2	8 ± 3^§^^¶^
Modified DF score	28 ± 25	24 ± 21	21 ± 7^‡^	54 ± 31^§^^¶^

Numbers are expressed as the mean ± standard deviation. ^†^ Numbers in parentheses are the percentage of patients in each group. ^‡^ *p* value < 0.05 comparing group 1 and group 2. ^§^ *p* value < 0.05 comparing group 1 and group 3. ^¶^ *p* value < 0.05 comparing group 3 and group 3. AD, acute decompensation; CLIF-SOFA, chronic liver failure-sequential organ failure assessment; DF, discriminant function; MELD, Model for End-Stage Liver Disease.

**Table 2:** Significant predictive factors for long-term mortality (including transplantation) in acutely deteriorated alcoholic LC patients.

	Univariate		Multivariate Model One		Multivariate Model Two	
	HR (95% C.I.)	*p*-Value	HR (95% C.I.)	*p*-Value	HR (95% C.I.)	*p*-Value
Male	0.89 (0.65–1.21)	0.446				
Age	1.00 (0.98–1.01)	0.657				
Previous AD	1.38 (1.08–1.76)	0.010	1.44 (1.13–1.85)	0.004	1.48 (1.16–1.90)	0.002
CTP score	1.50 (1.41–1.60)	<0.001	1.16 (1.07–1.25)	<0.001		
MELD score	1.14 (1.12–1.15)	<0.001			1.07 (1.04–1.09)	<0.001
CLIF -SOFA score	1.36 (1.31–1.41)	<0.001	1.30 (1.23–1.36)	<0.001	1.23 (1.16–1.30)	<0.001

AD, acute decompensation; C.I., confidence interval; CTP, Child-Turcotte-Pugh; CLIF-SOFA, chronic liver failure-sequential organ failure assessment; HR, hazard ratio; MELD, model for end-stage liver diseases.

**Table 3:** Comparison of the characteristics of patients with and without previous acute decompensation (AD) among groups 1 & 2.

	Total (*n* = 737)	No Previous AD (*n* = 362)	Previous AD (*n* = 375)	*p*-Value
Male	630 (85.5%)	313 (86.5%)	317 (84.5%)	0.457
Age	54 ± 9	54 ± 9	54 ± 10	0.691
Types of AD at admission				
Ascites	225 (30.5%)	133 (36.7%)	92 (24.5%)	<0.001
Hepatic encephalopathy	116 (15.7%)	39 (10.8%)	77 (20.5%)	<0.001
Gastrointestinal bleeding	361 (49.0%)	161 (44.5%)	200 (53.3%)	0.016
Infection	61 (8.3%)	30 (8.3%)	31 (8.3%)	1.000
CTP score	9 ± 2	9 ± 2	9 ± 2	0.877
MELD score	16 ± 6	16 ± 6	16 ± 6	0.610
CLIF-SOFA score	5 ± 3	5 ± 3	5 ± 3	0.151

AD, acute decompensation; CTP, Child-Turcotte-Pugh; CLIF-SOFA, chronic liver failure-sequential organ failure assessment; MELD, model for end-stage liver diseases.

**Table 4:** Relative risks of severe AH experience (group 3) compared with the risks in groups 1 & 2 in various subgroups according to survival and the presence of previous AD history.

	The Entire Cohort		Cohort Who Survived More Than Three Months		Cohort Who Survived More Than Six Months		Cohort Who Survived More Than 12 Months	
	HR (95% C.I)	*p*-Value	HR (95% C.I)	*p*-Value	HR (95% C.I)	*p*-Value	HR (95% C.I)	*p*-Value
The entire cohort	2.73 (2.09–3.57)	<0.001	1.95 (1.30–2.93)	0.001	2.03 (1.27–3.23)	0.003	2.13 (1.14–3.99)	0.018
No previous AD	3.18 (2.16–4.69)	<0.001	1.64 (0.85–3.20)	0.143	1.77 (0.85–3.71)	0.128	2.09 (0.84–5.20)	0.115
Previous AD	2.64 (1.80–3.88)	<0.001	2.60 (1.55–4.35)	<0.001	2.70 (1.48–4.93)	<0.001	2.67 (1.11–6.39)	0.022

AD, acute decompensation; C.I., confidence interval; HR, hazard ratio.

## Supplementary Materials

The following are available online at https://www.mdpi.com/2077-0383/8/10/1600/s1, Figure S1: scheme of cohort.
