# Supplementary figures and images for: The Impact of Previous Acute Decompensation on the Long-Term Prognosis of Alcoholic Hepatitis in Cirrhotic Patients
Source: J Clin Med. 2019 Oct 3;8(10):1600. doi: 10.3390/jcm8101600 (PMC6832392; doi:10.3390/jcm8101600)

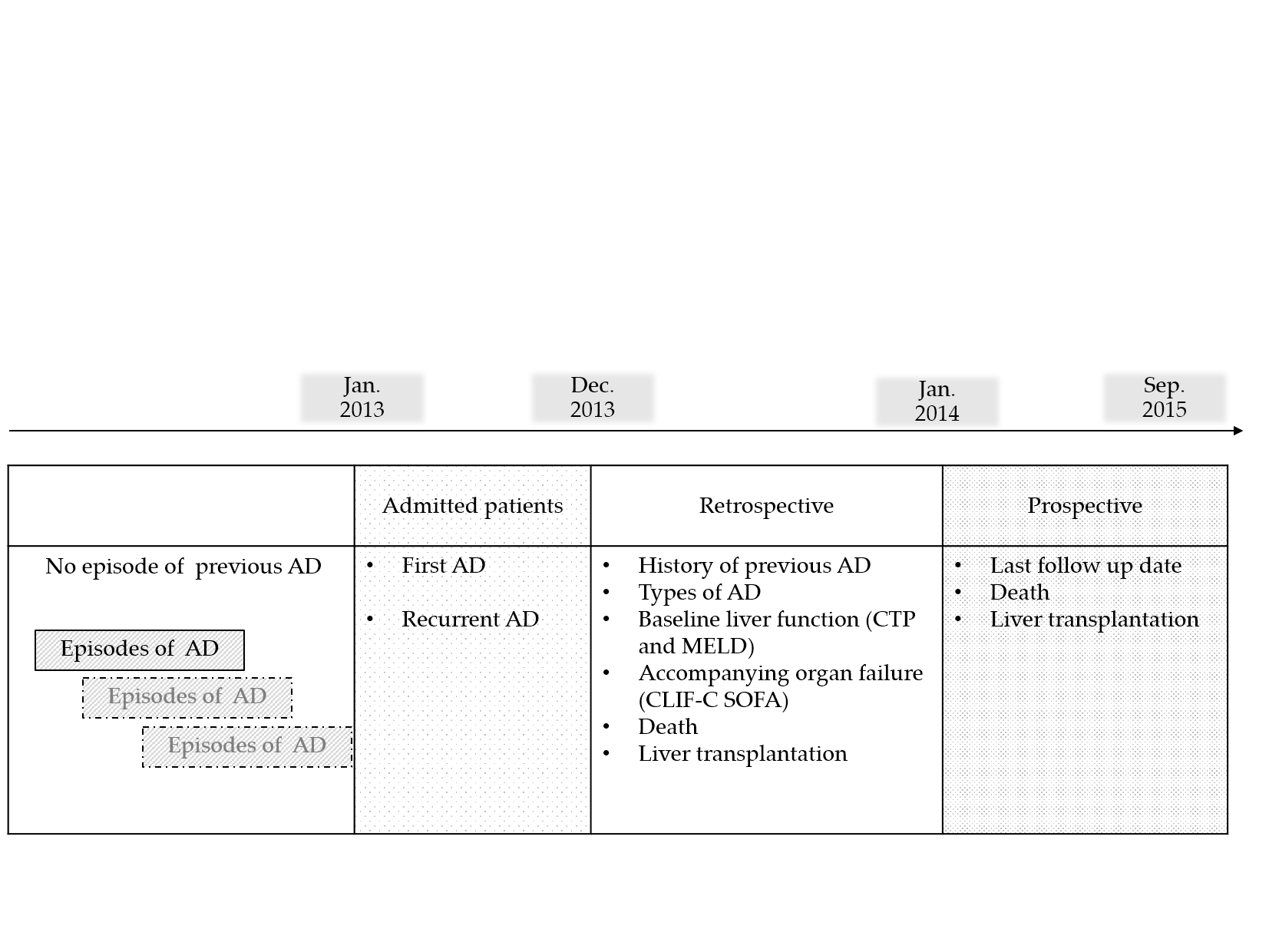

Supplement: Supplementary file 1 [file jcm-08-01600-s001.zip › jcm-589106-supplementary.tif]
